# Promotion of the resistance of human oral epithelial cells to herpes simplex virus type I infection via N6-methyladenosine modification

**DOI:** 10.1186/s12903-023-02744-2

**Published:** 2023-02-23

**Authors:** Junping Xu, Yuping Qi, Qi Ju

**Affiliations:** 1 Department of Stomatology, Beilun Branch of the First Affiliated Hospital of Zhejiang University, Ningbo, 315800 China; 2 Department of Oral Medicine, Institute of Stomatology, Qilu Hospital, Jinan, 250012 China; 3 Department of General Surgery, Beilun Branch of the First Affiliated Hospital of Zhejiang University, Ningbo, 315800 China

**Keywords:** Herpes simplex virus 1, m6A, alkb homolog 5, Fatty mass and obesity-associated gene, Oral herpes simplex, Type I interferon

## Abstract

**Objective:**

This study aimed to explore the mechanism behind N6-methyladenosine (m6A) modification of the total ribonucleic acid (RNA) involved in the resistance to herpes simplex virus type I (HSV-1) infection in oral epithelial cells.

**Method:**

The variation in m6A modification level on messenger RNA following HSV-1 infection was determined using the RNA dot blot method. The expression levels of alpha-ketoglutarate-dependent dioxygenase lab homolog 5 (ALKBH5) protein and fatty mass and obesity-associated genes (FTO) were determined using real-time fluorescence quantification polymerase chain reaction and the western blot technique, respectively. Next, after suppressing the expression of ALKBH5 or FTO via small interfering RNA, human immortalised oral epithelial cells (HIOECs) were infected with HSV-1, followed by measurement of the viral load or expression level of type I interferon (I-IFN) and interferon-stimulated genes (ISGs).

**Results:**

The m6A modification level was significantly increased following HSV-1 infection of the HIOECs (*P* < 0.05), while the expression of ALKBH5 and FTO genes was reduced (*P* < 0.01). Moreover, the suppression of ALKBH5 or FTO increased the production of I-IFN and ISGs during the HSV-1 infection of the HIOECs (*P* < 0.01), and the viral load was significantly reduced (*P* < 0.01).

**Conclusion:**

During oral HSV-1 infection, the m6A level was increased through the down-regulation of ALBHK5 and FTO expression, increasing I-IFN production and the promotion of HSV-1 clearing in HIOECs.

## Introduction

Herpes simplex is the most commonly found oral mucosal disease in oral clinics and largely manifests as recurrent herpetic stomatitis that is difficult to cure. Meanwhile, herpes labialis is one of the most prevalent orally transmitted diseases worldwide, one that can seriously affect the individual’s quality of life [1], while tobacco-associated oral lesions are highly prevalent in Asian countries [2]. Herpes simplex virus-1 (HSV-1) is the causative agent of herpes labialis [3] and establishes a life-long latent infection after invading the body. No effective vaccine is currently available [4]. After the virus infects the body, the body quickly recognises and responds through the innate immune system. The type-I interferon (I-IFN) signalling pathway is the first important line of defence of the host against HSV-1 [5]. Following HSV-1 infection, neurons secrete and respond to I-IFN, activating the Janus kinase/signal transducer and the transcription signalling pathway, promoting the expression of antiviral interferon-stimulated genes (ISGs) [6]. However, the molecular mechanism behind how the body regulates the antiviral immune response to resist HSV-1 infection remains unclear.

Epigenetics is currently a key research area in the field of basic medicine. This area of study mainly includes deoxyribonucleic acid (DNA) modification, ribonucleic acid (RNA) modification, histone modification and non-coding RNA, and contributes to the regulation of various physiological and pathological processes. Among these processes, N6-methyladenosine (m6A) modification is the most ubiquitous base modification for eukaryotic messenger RNA (mRNA) [7] and has the capacity for regulating mRNA splicing, transport, translation and degradation processes, and plays a key role in various physiological and pathological processes [8]. Studies have reported that RNA m6A modification plays an important role in the immune homeostasis of the host's innate or acquired immune response to pathogens [9]. The occurrence of RNA m6A modification is mediated via methyltransferase (METTL), including METTL 3 and METTL 14 proteins, and Wilms tumour 1-associated protein (WTAP) [10].

The RNA m6A modification process is a dynamic and reversible regulatory process that is dynamically regulated with the involvement of m6A writers (METTL3, METTL14 and WTAP) and readers (YTHDF1 and YTHDF2), and can impact many cellular processes and pathways. A recent study demonstrated that viruses could use m6A to ensure that their RNA avoids innate immune sensing. The removal of m6A modification is mediated by demethylases, including alpha (α)-ketoglutarate-dependent dioxygenase lab homolog 5 protein (ALKBH5) [11], and fat mass and obesity-associated protein (FTO) [12, 13], the latter being highly expressed in various tissue and organs of both adults and foetuses; the loss of FTO can lead to a significant increase in the level of mRNA m6A [14, 15].

Studies have found that the ALKBH5-mediated reduction of RNA m6A levels promotes the proliferation of cervical cancer cells, and increasing the m6A levels by inhibiting ALKBH5 may have anticancer effects [16]. However, there currently exists little research on ALKBH5 and FTO concerning antiviral infection.

In addition, a variety of reading proteins can specifically recognize m6A modification in eukaryotic cells, including YTHDC1, YTHDF1 and YTHDF2 [17–19], all of which can recognise m6A modification on RNA and determine the fate of its target genes.

As an important epigenetic regulation, RNA m6A modification has not been reported to participate in the body's antiviral innate immune response to HSV-1. In the present study, the human immortalised oral mucosal epithelial cell (HIOEC) line was selected as the in vitro research focus. Following virus infection, molecular biological assays were used to explore the function and mechanism of RNA m6A modification in the resistance of HIOECs to HSV-1 infection at the molecular and physiological levels [20].

## Materials and method

### Cell lines and viruses

The HIOEC line, the African green monkey kidney cell line (Vero) and the canine kidney cell line (MDCK) were all obtained from the American Culture Collection before HSV-1 was cultured and expanded with a Vero monolayer.

### Main reagents

Dulbecco's modified Eagle medium was purchased from PAN company, foetal bovine serum (FBS) was purchased from Gibco Co., and RNase inhibitor was purchased from Takara Co. A SYBR® Green Real-Time Polymerase Chain Reaction Master Mix fluorescence quantitative (RT-qPCR) kit and reverse transcription kit were purchased from Toyobo Co., a TRIzol™ reagent was purchased from Thermo Fisher Scientific Co. and an INTERFERin® transfection reagent was purchased from PolyPlus Co.

### Cell culture

The cells, frozen in liquid nitrogen, were removed and transferred into a 37 °C water bath. Then, 10-times the volume of the culture medium was added after thawing the cryopreservation solution, which was mixed well and centrifuged at 1000 rpm for 5 min before the supernatant was discarded and 10% FBS was added. The cells were then resuspended in a fresh medium of FBS, transferred to a cell culture flask and cultured in a 5% carbon dioxide cell incubator at a constant temperature of 37 °C, based on the method prescribed by Shanghai Mcellbank Biotechnology Co., Ltd. When the cells were 80%–90% confluent, they were digested using 0.25% trypsin-ethylenediaminetetraacetic acid and transferred to culture flasks or plating.

### Transfection of small interfering ribonucleic acid

The cell confluency was controlled to approximately 30%, and a pre-warmed fresh medium (containing 10% FBS) was used, with the instructions for the INTERFERin transfection followed. The virus amount was 5 × 10^3^, and the multiplicity of infection was 5. After 8 h of transfection, the pre-warmed fresh medium was changed, and subsequent experiments were carried out 48–72 h after transfection. The specific target sequences of human ALKBH5 and FTO were designed by GenePharma Company, and the sequences are presented in Table 1.

**Table 1:** ALKBH5, FTO interference specific target sequences

Target	Target sequence
ALKBH5 siRNA-1	5′-GAAAGGCTGTTGGCATCAATA-3′
ALKBH5 siRNA-2	5′-CCACCCAGCTATGCTTCAGAT-3′
ALKBH5 siRNA-3	5′-CCTCAGGAAGACAAGATTAGA-3′
FTO siRNA-1	5′-CGGTTCACAACCTCGGTTTAG-3′
FTO siRNA-2	5′-TCGCATGGCAGCAAGCTAAAT-3′
FTO siRNA-3	5′-ACCTGAACACCAGGCTCTTTA-3′
NC siRNA	5′-GAAUACGUACCCCAUUAUATT-3′

### Total ribonucleic acid extraction

The TRIzol-based method for extracting the total cell RNA involved extracting the total RNA according to the TRIzol reagent instructions, dissolving the RNA in diethyl pyrocarbonate and using a Nanodrop-2000 instrument to determine the RNA purity and concentration before the solution was stored at − 80 °C for later use.

### Real-time quantitative polymerase chain reaction

The RNA was extracted using the TRIzol reagent, and 1 μg of RNA was taken and reverse transcribed using the above-noted kit to obtain complementary DNA (cDNA). Using the cDNA as a template, gene quantitative primers were added, and the amplification reaction was performed using a qPCR instrument (Roche Lightcycler 40) according to the manufacturer’s instructions. Using the 2^−ΔΔCt^ method, the relative expression levels of the genes to be tested were calculated using beta-actin as an internal reference. And the relative expression level of indicated genes was normalized to β-actin. And the negative control was set to a value of 1. The primer sequences for the fluorescence qPCR procedure are shown in Table 2.

**Table 2:** Primers for quantitave real-time PCR amplification

Gene		Sequence(5′ → 3′)
Human β-actin	F	CTGGAACGGTGAAGGTGACA
	R	AAGGGACTTCCTGTAACAATGCA
Human ALKBH5	F	CGGCGAAGGCTACACTTACG
	R	CCACCAGCTTTTGGATCACCA
Human FTO	F	TTCATGCTGGATGACCTCAATG
	R	GCCAACTGACAGCGTTCTAAG
Human IFN-α	F	GCCTCGCCCTTTGCTTTACT
	R	CTGTGGGTCTCAGGGAGATCA
Human IFN-β	F	GCTTGGATTCCTACAAAGAAGCA
	R	ATAGATGGTCAATGCGGCGTC
Human ISG15	F	CGCAGATCACCCAGAAGATCG
	R	TTCGTCGCATTTGTCCACCA
Human CXCL10	F	GTGGCATTCAAGGAGTACCTC
	R	TGATGGCCTTCGATTCTGGATT

### Dot blot analysis of ribonucleic acid N6-methyladenosine methylation status

The RNA m6A methylation was analysed using the dot blot method. In brief, the total RNA was isolated using the TRIzol-based method before the total RNA concentration and purity were measured using the Nanodrop-2000 instrument. The RNA was denatured via heating at 72 °C for 5 min before direct cooling on ice. Next, the RNA (50–100 ng) was spotted directly on a positively charged nylon membrane (Pall) and air-dried for 5 min. The membranes were then ultraviolet (UV)-crosslinked using a UV-crosslinker, blocked with 5% non-fat milk in a Tris-buffered saline–polysorbate 20 solution and incubated with an anti-m6A antibody (Synaptic Systems) overnight at 4 °C. The horseradish peroxidase-conjugated anti-rabbit immunoglobulin secondary antibody was added to the membrane for 1 h at room temperature with gentle shaking and then developed with enhanced chemiluminescence. Methylene blue staining was used as a control, and the chemiluminescence imager was finally exposed.

### Determination of virus titers

Inoculate MDCK cells were inoculated into a 96-well culture plate; the supernatant of the cells to be tested was serially diluted, and 10^−1^, 10^−2^, 10^−3^, 10^−4^, 10^−5^, 10^−6^, 10^−7^, 10^−8^, 10^−9^, 10^−10^ and other different dilution gradients were obtained. Eight wells of each dilution plate were seeded and, after 3 days, the cytopathic conditions were observed and recorded, and the median tissue culture infectious dose (TCID50) was calculated.

### Western blot procedure

The collected cells were washed twice with a phosphate-buffered saline solution before a radioimmunoprecipitation assay lysate containing 1% phenylmethylsulfonyl fluoride and a 2% phosphatase inhibitor was added and cryo-milled using a high-throughput tissue homogeniser. The homogenate was then centrifuged at 4 °C and 12,000 rpm for 20 min and the supernatant was aspirated. The protein concentration of the supernatant was determined using a bicinchoninic acid kit according to the manufacturer's instructions. Equal amounts of protein from each sample were then separated using 12% sodium dodecyl sulphate–polyacrylamide gel electrophoresis and transferred to polyvinylidene fluoride membranes. The blots were cut prior to hybridisation with antibodies during blotting. Following incubation with primary and secondary antibodies and washing, the protein bands were detected using the Tanon Gel Imaging System.

### Statistical processing

All the experimental results were obtained from three or more independent repeated experiments, with the results expressed in terms of mean ± standard deviation. The statistical analysis was conducted using the SPSS Statistics 17.0 software program, and an independent sample t-test was adopted to analyse the difference between the two independent samples. The one-way analysis of variance method and the least-significant difference t-test were used for the multiple comparison analyses of more than three groups of independent samples. A *P* value of < 0.05 indicated that the difference was statistically significant.

## Results

1. Change in ribonucleic acid N6-methyladenosine modification following herpes simplex virus type I infection of oral epithelial cells

**Fig. 1:**
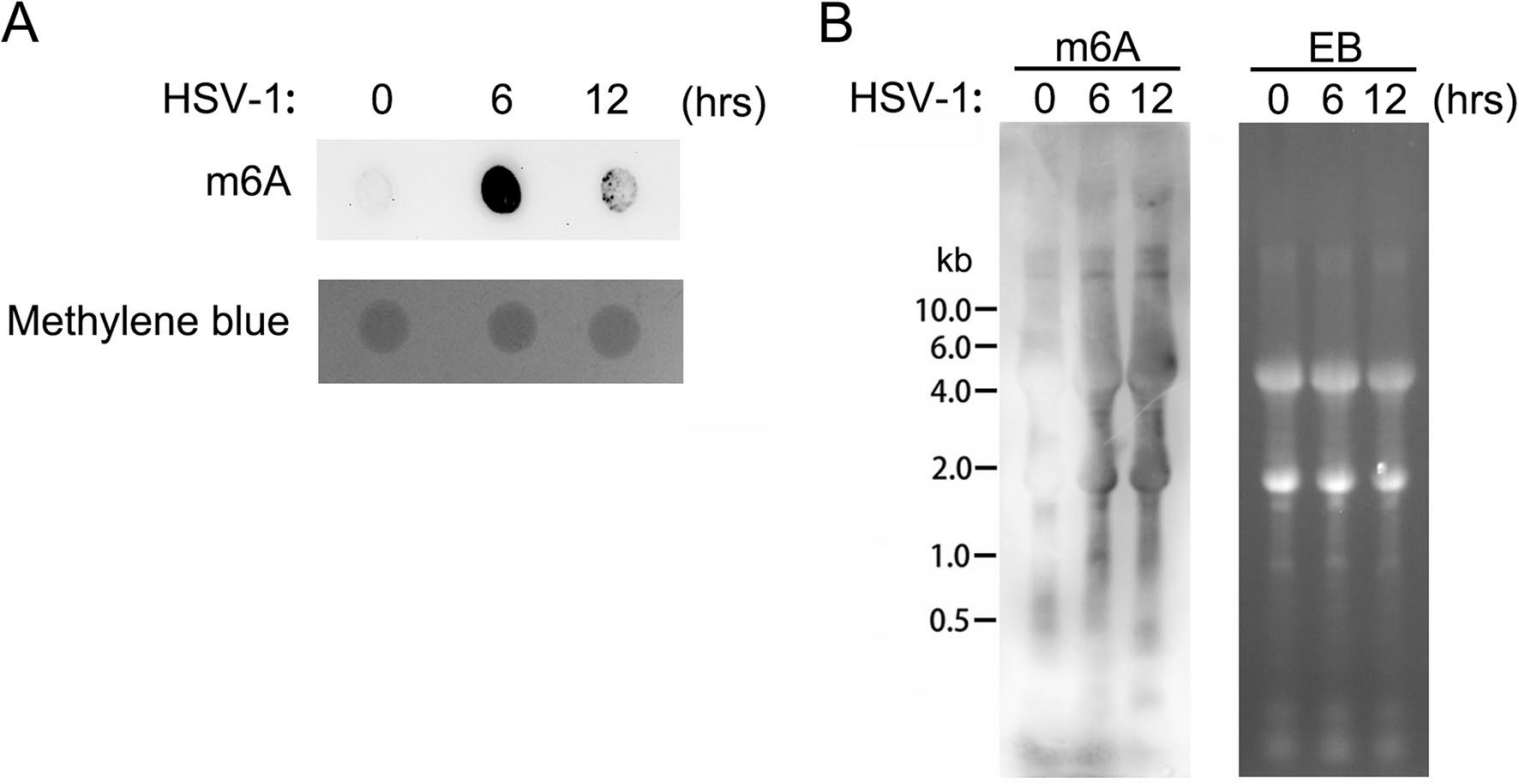
HSV-1 infection leads to increased RNA m6A levels in oral epithelial cells. **A** Dot blot was used to detect the changes of m6A levels in total RNA of HIOEC cells after HSV-1 infection. **B** Gel electrophoresis was used to detect changes in m6A levels of total RNA from HIOEC cells following HSV-1 infection. *Note*: “EB” Ethidium Bromide, it usually used to quantify the RNA/DNA

To explore whether RNA m6A modification was involved in HSV-1 infection, the HIOEC lines were first infected with HSV-1 before the dot blot method was used to detect any changes in the m6A level of the total cellular RNA. The results showed that the intracellular m6A levels were significantly increased following HSV-1 infection of the HIOECs (Fig. 1), which suggested that RNA m6A modification may have been involved in regulating the innate immune response against HSV-1 infection.

2. The expression of alpha-ketoglutarate-dependent dioxygenase lab homolog 5 protein and fatty mass and obesity-associated genes in oral epithelial cells following herpes simplex virus type I infection

**Fig. 2:**
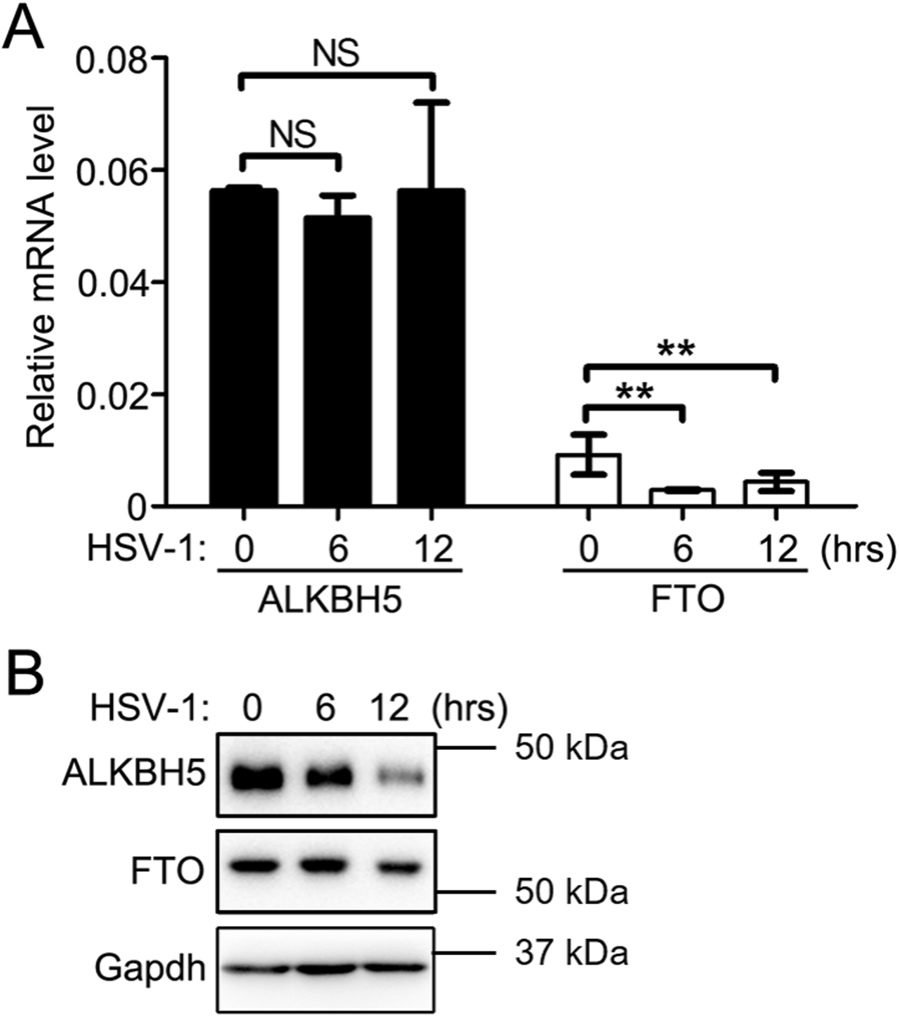
HSV-1 infection down-regulated the expressions of ALKBH5 and FTO in oral epithelial cells. **A** Changes in mRNA levels of ALKBH5 as well as FTO protein in HIOEC cells following HSV-1 infection. **B** Changes in protein levels of ALKBH5 as well as FTO protein in HIOEC cells following HSV-1 infection. **P < 0.01

As noted, ALKBH5 and FTO proteins act as demethylases to mediate the removal of m6A modifications [10], thereby regulating the intracellular m6A levels. In this study, it was assumed that up-regulation of the m6A level following HSV-1 infection may have been related to ALKBH5 and FTO proteins. To test this hypothesis, the expression levels of ALKBH5 and FTO proteins and mRNA were determined after infecting the HIOEC lines with HSV-1 (see Fig. 2 for these results). Following HSV-1 infection of the HIOECs, the mRNA expression level of the ALKBH5 gene did not change significantly (*P* > 0.05, Fig. 2A), but the protein level was significantly down-regulated (Fig. 2B). Concurrently, the mRNA expression level of the FTO gene was significantly reduced (*P* < 0.01, Fig. 2A), as was the protein expression level (Fig. 2B). These results indicated that, following the HSV-1 infection of the cells, the host cells reduced the level of ALKBH5 protein by inhibiting the translation link of the ALKBH5 gene or promoting its protein degradation, subsequently down-regulating the level of FTO protein by inhibiting the transcription level of the FTO gene or promoting mRNA degradation, ultimately leading to elevated levels of m6A modification of the RNA in the host cells.

3. The increase in type I interferon expression via alpha-ketoglutarate-dependent dioxygenase lab homolog 5 protein and fatty mass and obesity-associated gene interference

**Fig. 3:**
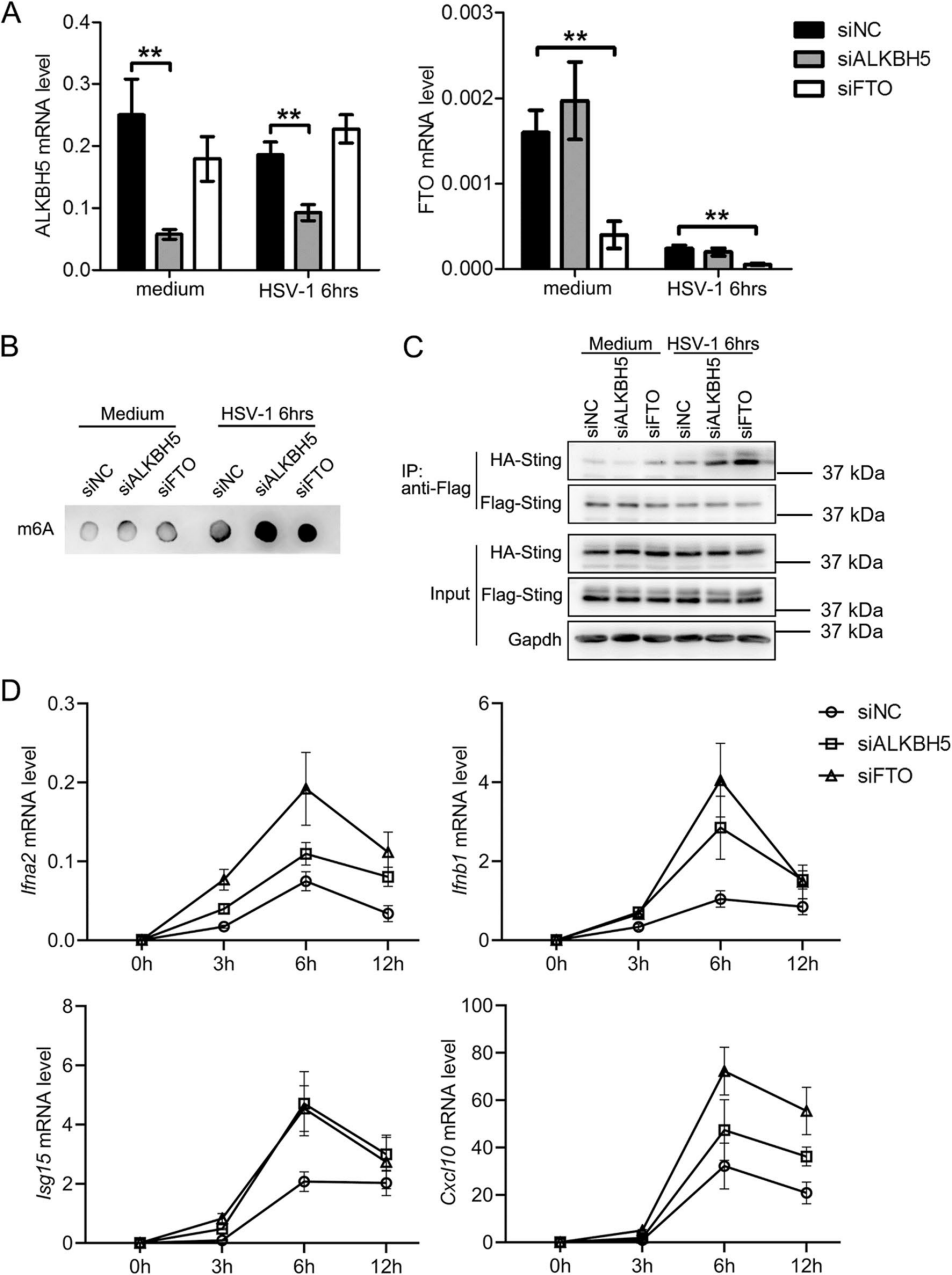
Interfering with ALKBH5 or FTO can promote HSV-1-induced expression of type I interferon. **A** Knockdown of ALKBH5 and FTO using siRNA in HIOEC cells. **B** Knockdown of ALKBH5 and FTO increased m6A modification of RNA in HIOEC cells. **C** Knockdown of ALKBH5 and FTO activated the STING signaling pathway in HIOEC cells. **D** Knockdown of ALKBH5 and FTO increased mRNA expression levels of IFNα, IFNβ, ISG15 and CXCL10 in HIOEC cells. **P < 0.01

Following virus infection, the body's natural immune system can respond immediately, synthesising and secreting a large amount of I-IFN and ISG and implementing an antiviral function [5]. Therefore, to explore whether the RNA m6A modification mechanism mediated the process of cell secretion and the synthesis of I-IFN and ISG and whether it participated in the antiviral response, small interfering RNA technology was used to silence the expression of ALKBH5 and FTO genes in the HIOECs before the cells were infected with HSV-1, and the RNA m6A modification level and I-IFN (including IFNα, and IFN beta[β]) and ISG (including ISG15 and CXCL10) expression were determined. It has been reported that ISG15 and CXCL10 genes were both related to the immune system of the host following HSV infection. The results indicated that the silencing of ALKBH5 and FTO gene expression (*P* < 0.01, Fig. 3A) could significantly increase the m6A modification level of cellular RNA (Fig. 3B) and activate the STING signaling (Fig. 3C), while the expressions of IFNα, IFNβ, ISG15 and CXCL10 were also significantly up-regulated (*P* < 0.01, Fig. 3D). This suggests that the body may regulate the synthesis and secretion of I-IFN and ISG by up-regulating the m6A level of RNA in oral cells, ultimately participating in the immune response of cells against HSV-1 infection.

4. Promoting the clearance of herpes simplex virus type I via alpha-ketoglutarate-dependent dioxygenase lab homolog 5 protein and fatty mass and obesity-associated gene interference

**Fig. 4:**
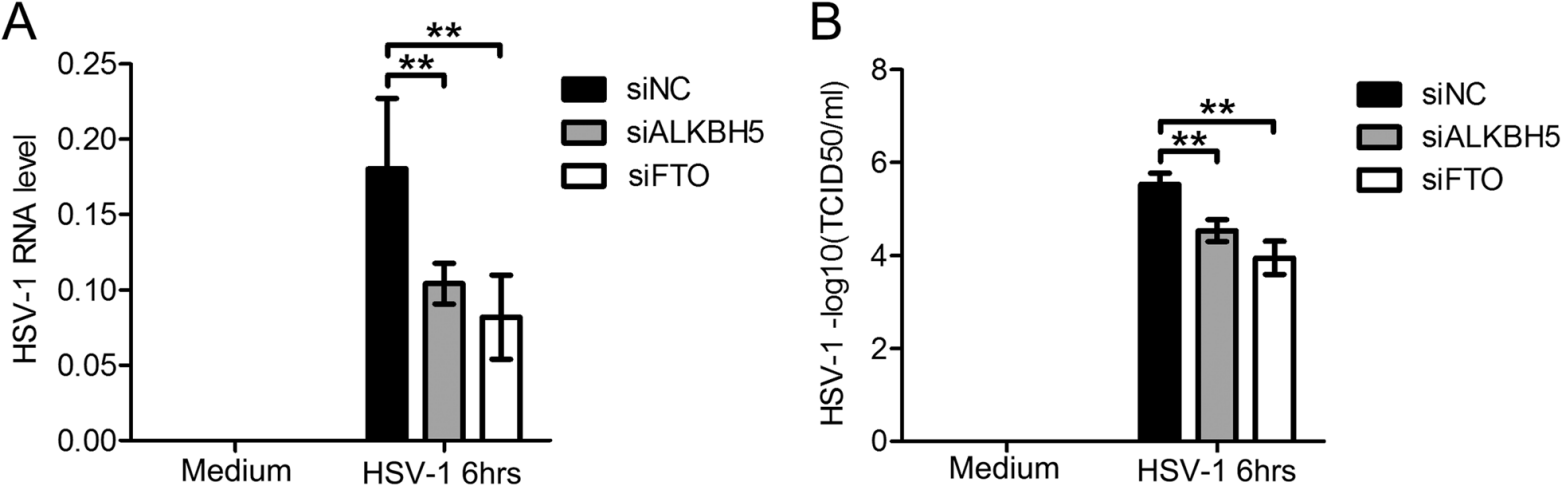
Interfering with ALKBH5 or FTO reduces intracellular HSV-1 load. **A** Intracellular viral RNA levels decreased after knockdown of ALKBH5 and FTO. **B** Intracellular viral load decreased after knockdown of ALKBH5 and FTO. **P < 0.01

To further confirm that ALKBH5 and FTO gene silencing could promote the resistance of HIOECs to HSV-1 infection, the level of intracellular viral RNA and the viral load in the cell culture supernatant was determined. The results indicated that when the ALKBH5 and FTO genes were suppressed, both the intracellular viral RNA level and the viral load in the cell culture supernatant decreased, and the difference was statistically significant (*P* < 0.01, Fig. 4A, B). Therefore, it was preliminarily concluded that the down-regulation of ALKBH5 or FTO gene expression could improve the immune function of oral epithelial cells against HSV-1 infection.

## Discussion

The HSV-1 virus is the most common oral infection virus and the most host-susceptible virus in humans. According to statistics, 45%–98% of the world's population has been infected with the HSV-1 virus, often resulting in oral herpes simplex [21]. Recent studies have found that HSV-1 infection is, in fact, closely related to the incidence of Alzheimer's disease [22]. The innate immune system is the body's first line of defence against infection by pathogenic microorganisms. When the virus invades the body, the mucosal, epithelial and innate immune cells (including mononuclear macrophages and dendritic cells) can recognise the virus and activate the innate immune response, secreting a large amount of I-IFN and promoting ISG expression to achieve the immune effect of inhibiting virus proliferation, eliminating the virus and thus terminating any risk of infection [23]. Accordingly, there is clinical value in studying how the human innate immune system resists HSV-1 virus infection.

Since 2012, RNA modification has rapidly developed into an important international research focus, with m6A modification the most commonly used type of base modification on eukaryotic mRNA and playing an important role in physiological and pathological processes, such as potency, cell differentiation and tumour development [24, 25]. While the functional study of m6A modification in oral clinical medicine has rarely been conducted, the experiments conducted by Xin Lou et al. [8] revealed that m6A modification played an important role in the signalling network of the immune evasion mechanism, as well as in innate and adaptive immunity during disease progression. In this context, molecular tachyphylaxis plays a fundamental role as it can cause certain proteins to suppress or affect the implementation of m6A, which suggests that m6A modification may be involved in the immune response of oral cells to antiviral infection.

The in vitro experiments in the current study revealed that the m6A modification level of the total cell RNA was significantly increased after the HIOEC line was infected with HSV-1, indicating that m6A modification is involved in regulating the immune response process of oral cells against HSV-1 infection.

Current studies have demonstrated that m6A modification acts as a dynamic and reversible process, with the demethylases, ALKBH5 and FTO mediating m6A modification removal [26]. The deletion of FTO can lead to significant upregulation of mRNA m6A levels [14], while Zhang et al. [27] demonstrated that the overexpression of ALKBH5 could reduce the methylation level of the target gene, NANOG mRNA.

In the current study, to reveal the mechanism behind the elevated m6A level when HIOECs were infected with HSV-1, the expression levels of the ALKBH5 and FTO proteins were determined, with the results indicating that the expression of ALKBH5 and FTO in the cells decreased significantly following infection. It was speculated that the FTO transporter had been down-regulated, which may have been due to the inhibition of the transporter expression.

It was also speculated that, following HSV-1 infection in the oral cavity, the body could increase the m6A modification of the downstream gene RNA by inhibiting the expression of ALKBH5 and FTO proteins and that its secretion was affected by the expression of transporters, promoting the antiviral innate immune response and resisting HSV-1 infection. However, the specific mechanisms behind the regulation of the host cell signalling were not clear, and further investigation is required in this regard.

The m6A-modified demethylases, ALKBH5 and FTO, were able to mediate the expression of specific target genes and participated in multiple physiological responses. Li et al. [28] found that FTO regulated the expression of targets such as ASB2 and RARA by reducing the m6A levels in the mRNA transcripts of the target genes, thereby enhancing leukemic oncogene-mediated cell transformation and leukemogenesis. Elsewhere, it was found that ALKBH5 could affect a mouse’s reproductive ability [29] or tumour progression [30] by regulating the m6A level of the downstream target genes. The expression of I-IFN and downstream ISG is involved in the body's innate immune response against viral infection. Accordingly, it was speculated that the m6A modification mediated by ALKBH5 and FTO may be able to regulate the expression of I-IFN and ISG.

Expression plays a role in oral cell resistance to the HSV-1 virus. In this study, the expression of the antiviral effectors, I-IFN and ISG, was determined by silencing the expression of ALKBH5 and FTO genes, with the results indicating that the silencing of these genes could significantly promote the expression of I-IFN and ISG. Furthermore, after determining the HSV-1 loading in the host cells and cell culture supernatants, it was found that both ALKBH5 and FTO gene silencing could significantly promote virus clearance. As such, RNA m6A modification was clearly involved in the immune response of oral cells against HSV-1 infection, which was mediated by ALKBH5 and FTO, and ultimately promoted virus clearance in the infected cells by regulating the expression of I-IFN and ISG. The results of this study imply that ALKBH5 and FTO could be used as potential clinical therapeutic targets. As such, this study has important scientific value and clinical significance.

However, the study also had certain limitations. First, the research mainly relied on in-vitro cells as the research model and thus lacked data support from in-vivo experiments. Second, only the m6A modification level of the overall RNA was determined to explain the subsequent conclusion, rendering the mechanism repetitive and one-sided and lacking the study of m6A modification of specific target genes.

In terms of future research, first, regarding the specific mechanisms, it is speculated that the increased m6A modification of downstream gene RNA results in the down-regulation of many genes related to important cell processes following HSV infection. Further research into the specific mechanism of m6A modification in the anti-HSV-1 infection of oral cells will also be conducted.

## Conclusions

In summary, this study revealed that the m6A modification level of RNA in epithelial cells was increased by reducing the expression levels of the demethylases, ALKBH5 and FTO, via promoting the antiviral immune responses. A new mechanism of the body's resistance to HSV-1 virus infection was also revealed. Overall, the study provides a theoretical basis for the clinical treatment of oral herpes simplex.

## Data Availability

All data generated or analyzed during this study are included in this published article.
